# Computational Approaches and Use of Chiroptical Probes in the Absolute Configuration Assignment to Natural Products by ECD Spectroscopy: A 1,2,3-Trihydroxy-*p*-menthane as a Case Study

**DOI:** 10.3390/biom12030421

**Published:** 2022-03-09

**Authors:** Giulia Marsico, Umberto Calice, Patrizia Scafato, Sandra Belviso, Antonio Evidente, Stefano Superchi

**Affiliations:** 1 Department of Sciences, University of Basilicata, Via dell’Ateneo Lucano 10, 85100 Potenza, Italy; giuliamar86.gm@gmail.com (G.M.); umberto.calice@studenti.unibas.it (U.C.); patrizia.scafato@unibas.it (P.S.); sandra.belviso@unibas.it (S.B.); 2 Department of Chemical Sciences, University of Naples “Federico II”, Complesso Universitario Monte Sant’Angelo, Via Cintia 4, 80126 Napoli, Italy; antonio.evidente@unina.it

**Keywords:** hydroxy menthane, chiral natural products, absolute configuration, biphenyls, chiroptical probes, electronic circular dichroism

## Abstract

In this study, the computational analysis of electronic circular dichroism (ECD) spectra and the employment of biphenyl chiroptical probes were compared in the absolute configuration assignment of (-)-1α,2α,3β-trihydroxy-*p*-menthane (**1**), taken as a representative example of a UV-transparent chiral natural product. The usefulness of chiroptical probes in the configurational assignments of natural products and their complementarity to the computational protocols is herein highlighted. The biphenyl probe approach proves to be straightforward, reliable, and suitable for conformationally mobile and ECD silent compounds, not treatable by computational analysis of chiroptical data.

## 1. Introduction

The assignment of absolute configuration (AC) is a fundamental task to be addressed in the structural characterization of chiral compounds, including natural products. In fact, the key role of AC on the bioactivity of naturally occurring chiral metabolites [1,2], agrochemicals [3], or drugs [4,5] has been clearly established. It follows that knowledge of AC is a prerequisite for any study on either the biosynthesis or biological activity of natural products. Several approaches are available to determine the AC of chiral compounds; however, each one presents specific advantages and disadvantages [6]. For example, the classical chemical correlation or total synthesis is a very time-consuming approach, while X-ray diffraction [7] is not of general applicability. In fact, natural products are often available in tiny amounts and/or in non-crystalline form, i.e., features that prevent a direct assignment of AC by X-ray diffraction analysis [8]. For these compounds, the spectroscopic methods based on nuclear magnetic resonance (NMR) and chiroptical spectroscopy provide powerful tools for the AC assignment, allowing the treatment of molecules in solution. However, NMR methods [9] are essentially semi-empirical and are mainly employed for carbinols AC assignment [10], while chiroptical spectroscopies [11,12], such as optical rotation (OR), electronic circular dichroism (ECD), and vibrational circular dichroism (VCD), provide more general, nonempirical, sensitive, and reliable approaches for AC assignment of natural products. In recent years, the advance of computational protocols for the ab initio predictions of chiroptical properties [13,14] made this approach the method of choice for the AC assignment of chiral natural products [15,16]. However, the computational protocols can be impractical when treating natural products endowed with high conformational mobility and/or low chiroptical responses. In fact, these cases require extensive time-consuming computational conformational analyses and the use of two or more combined chiroptical spectroscopies to obtain reliable results [17,18]. A feasible and reliable alternative to the computational approaches is provided by the so-called “chiroptical probes”, which are particularly useful for the treatment of highly flexible molecules and require simpler spectra analysis that is within the reach of researchers who lack advanced expertise in spectroscopy and computations [19]. The term “chiroptical probe” defines achiral chromophoric moieties which, when linked to a chiral nonracemic substrate, give rise to diagnostic chiroptical signal(s), usually of the ECD spectrum, from which the AC of the substrate can be determined [20,21,22]. Although many chiroptical probes have been described so far, very few examples report their application to natural products of unknown AC [23,24]. Only very recently we have shown that 2,2′-bridged biphenyls, introduced by us some years ago for the AC assignment of chiral *threo* [25] and *erythro* [26] diols, carboxylic acids [27,28,29], and primary amines [30,31], also provide a versatile probe for the AC assignment of complex natural products [32,33]. In particular, we applied the biphenyl approach to assign AC to natural phytotoxins bearing a *threo* diol moiety [32] or a chiral acid group [33].

**Figure 1:**
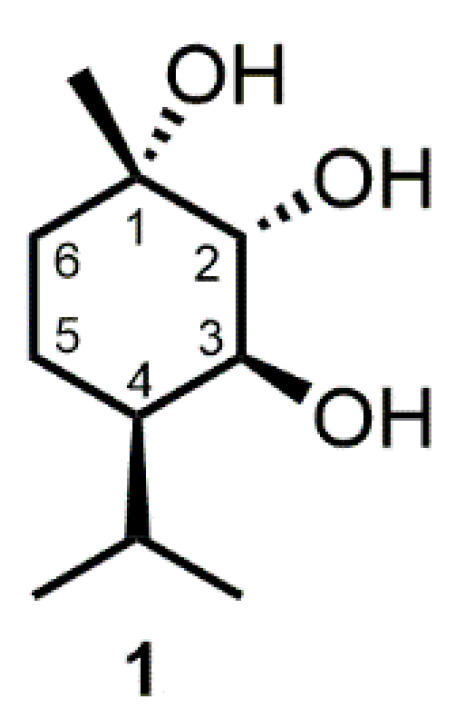
Structure of (-)-(1*S*,2*R*,3*S*,4*R*)-trihydroxy-*p*-menthane (**1**).

Our aim herein is to verify the reliability of the above method with naturally occurring *erythro* diols with weak UV absorption and, consequently, very low ECD response. Therefore, the biphenyl approach was tested in the AC assignment of the monocyclic monoterpene (-)-1α,2α,3β-trihydroxy-*p*-menthane (**1**) (Figure 1), taken as a suitable test compound. In fact, this aliphatic cyclic triol was expected to display weak UV and ECD signals in far-UV; thus, its AC assignment by ECD analysis was quite challenging. This compound bore three hydroxy groups with different relative configurations. In fact, OH on C-1 and C-2 were on the same side of the ring in the *erythro* configuration, while OH on C-2 and C-3 were on opposite sides and in a *threo* arrangement. Therefore, the possibility of checking the chemoselectivity of the derivatization with the biphenyl dioxolane was also provided. A comparison between the computational approach and the use of the biphenyl probe in the AC assignment of this natural product was also carried out. 

This natural monoterpene also presents a peculiar structure, which makes it an interesting natural fungal metabolite per se. In fact, simple monoterpenes are quite rare as fungal metabolites and, to the best of our knowledge, **1** is the only example of naturally occurring 1,2,3 trihydroxy-*p*-menthane derivatives reported so far. Compound **1** was first isolated from the culture filtrates of *Phomopsis* (syn. of *Fusicoccum*) *amygdali* together with a plethora of fusiccocanes diterpenoids [34], and further studies by isotope incorporation were able to elucidate its biosynthetic pathway [35]. The fungus also produced, as its main phytotoxin, the well-known fusicoccin, an α-glucoside of a carbotricyclic diterpene causing almond and peach canker disease, which is by far one of the most studied fungal phytotoxins, being the main topic of more than 1800 scientific publications. In the last two decades, the interest in fusicoccin has increased, as its target in animals is a 14-3-3 protein also involved in some human diseases and, in particular, in malignant cancer [36]. Preliminary investigations showed that **1** is essentially nontoxic to tomato and radish plants [37]; however, in that study, the low amount of **1** available from the culture filtrates of the fungus did not allow further investigation into its biological activity. Quite recently, **1** was also isolated from the endophytic fungus *Diaporthe* cf. *heveae*, together with a number of dihydroisocumarins, and was tested against *Phyllosticta citricarpa* and *Colletotrichum abscissum* fungi, as well as against some human cancer cell lines, showing no activity [38]. Besides **1**, the only other trihydroxy *p*-menthanes isolated from fungal sources are (-)-(1*R*,2*R*,4*R*,8*S*)-*p*-menthane-2,8,9-triol and its 8-epimer, which were isolated from *Flammulina velutipes* [39], and (+)-(1*S*,2*S*,4*S*)-trihydroxy-*p*-menthane, which was isolated from the endophytic fungus *Phomopsis* sp. [40,41] and from *Diaporthe* sp. SXZ-19, an endophytic fungal strain of *Camptotheca acuminate* [42]. The latter hydroxy terpene was also isolated from plant sources, first from the pyrolytic products of the gum resin of *Boswellia carteri Birdw*. (incense “Aden”) [43], then from volatile oil of *Zanthoxylum budrunga* fruits [44], from the Chilean shrub *Luma gayana* [45], and from the seeds of *Trachyspermum roxburghianum*, a flowering herb belonging to the family of Apiaceae (Umbelliferae) and diffused in South Asia, South-East Asia, and Indonesia [46].

The relative configuration of **1**, previously assigned on the basis of ^1^H NMR data, was confirmed by the total synthesis of racemic **1** together with six stereoisomers [47], while its AC was later established to be (-)-(1*S*,2*R*,3*S*,4*R*) by GC chiral chromatography, comparing the retention time of the natural compound with that of its enantiomer (+)-**ent-1**, which was synthesized from (+)-limonene oxide [48]. However, this assignment method was quite time-consuming, requiring five synthetic steps to obtain (+)-**ent-1** from limonene oxide and, relying on the chromatographic comparison of the two enantiomers, which differ by a few seconds in retention time, was error-prone. Therefore, an independent approach to establish the AC of (-)-**1** appears advisable to reach a more reliable configurational assignment.

## 2. Materials and Methods

### 2.1. General Experimental Procedure

Optical rotations were measured in a CH_3_Cl solution on a Jasco (Tokyo, Japan) P-1010 digital polarimeter. The UV and ECD spectra were recorded at room temperature on a Jasco (Tokyo, Japan) J815 spectropolarimeter, using 0.1 mm cells and concentrations of about 1 × 10^−^^3^ M in CH_3_CN solution. During measurement, the instrument was thoroughly purged with N_2_. ^1^H NMR spectra were recorded at 400 MHz in CDCl_3_ on either Bruker (Karlsruhe, Germany) or Varian (Palo Alto, CA, USA) Inova spectrometers. Analytical and preparative thin-layer chromatography (TLC) was performed on silica gel (Kieselgel 60, F_254_, Merck, Darmstadt, Germany) 0.25 mm and 0.5 mm plates, respectively. The compounds spots were visualized on TLC plates by exposure to UV light and/or by spraying them, first with 10% H_2_SO_4_ in MeOH, and then with 5% phosphomolybdic acid in EtOH, followed by heating at 110 °C for 10 min. Column chromatography (CC) was carried out on silica gel (Kieselgel 60, 0.063–0.200 mm, Merck, Darmstadt, Germany). The dimethyl acetal **2** was obtained as previously described [25,32]. CHCl_3_ was distilled from P_2_O_5_ prior its use and stored under nitrogen atmosphere. All solvent and reagents were purchased from Merck (Darmstadt, Germany) and used without further purification unless otherwise stated.

### 2.2. Production, Extraction and Purification of 1α,2α,3β-Trihydroxy-p-menthane (1)

From culture filtrates of *F. amygdali* grown to produce fusicoccin, (-)-lα,2α,3β-trihydroxy-*p*-menthane (**1**) was isolated. The extraction of the fungal culture filtrate [49] and the purification of the corresponding organic extract were carried out as previously reported [34], providing pure (-)-**1**. The ^1^H NMR spectrum of (-)-**1** recorded in CDCl_3_ (see Figure S1 in the Supplementary Material) was identical to that previously reported [34,48]. The purity of **1** > 98% was ascertained by ^1^H NMR and HPLC analyses: ^1^H NMR (400 MHz, CDCl_3_) δ 4.11 (m, 1H), 3.51 (d, *J* = 4.8 Hz, 1H), 2.7 (d, *J* = 3.0 Hz, 1H), 1.9 (br s, 1H), 1.6–1.8 (m, 4H), 1.5–1.4 (m, 2H), 1.4–1.5 (m, 1H), 1.38 (s, 3H), 1.02 (d, *J* = 6.8 Hz, 3H), 0.95 (d, *J* = 6.8 Hz, 3H). 

### 2.3. Synthesis of Biphenyl Dioxolane 3

To a solution of the triol (-)-**1** (1.4 mg, 7.4 μmol) in anhydrous CHCl_3_ (0.1 mL), the dimethylacetal **2** (2.5 mg, 10.0 μmol), 4 Å molecular sieves, and a crystal of *p*-toluene sulfonic acid were added. The mixture was stirred at room temperature overnight. The reaction mixture was monitored by TLC analysis, and when complete transformation of compound **1** was observed, the mixture was filtered and the solvent evaporated until dry. After chromatographic purification on silica gel (diethyl ether/petroleum ether 3:1 *v*/*v*), compound **3** was recovered as a glassy solid. ^1^H NMR (400 MHz, CDCl_3_) δ 7.42 (t, *J* = 8.6 Hz, 2H), 7.36−7.22 (m, 6H), 4.38 (m, 1H), 3.90 (d, *J* = 4.8 Hz, 1H), 2.95–2.70 (m, 4H), 1.65–1.35 (m, 6H), 1.43 (s, 3H), 0.99 (d, *J* = 7.0 Hz, 3H), 0.90 (d, *J* = 7.0 Hz, 3H).

### 2.4. Computational Details

Preliminary conformational analyses were performed with the Spartan02 (Wavefunction Inc., Irvine, CA, USA, version 02) package [50] by employing a MMFF94s molecular mechanics force field with Monte Carlo searching and arbitrarily fixing AC (1*S*,2*R*,3*S*,4*R*) for **1**. All possible conformers were searched, considering the degrees of freedom of the system within an energy window of 10 kcal/mol. The minimum energy conformers found by molecular mechanics were further fully optimized by the Gaussian09 (Gaussian, Inc., Wallingford, CT, USA, revision A. 02) package [51], and the density functional theory (DFT) was used at the DFT/B3LYP/6-31G(d) level, taking into account the solvent effect of CH_3_CN with the IEFPCM implicit model [52]. All conformers were real minima, no imaginary vibrational frequencies were found, and the free energy values were calculated and used to obtain the Boltzmann population of conformers at 298.15 K. For each conformer, UV and ECD spectra were calculated at the TDDFT/CAM-B3LYP/aug-cc-pVDZ/IEFPCM(CH_3_CN) level of theory, taking into account the first 30 excited states. The computed UV and ECD spectra were obtained as an average over the conformers Boltzmann populations. The ECD spectra were obtained by the Spec Dis package [53] from calculated excitation energies and rotational strengths, as a sum of Gaussian functions centered to the wavelength of each transition, with a parameter σ (width of the band at ½ height) of 0.4 eV.

## 3. Results and Discussion

### 3.1. Absolute Configuration Assignment by Computation of ECD Spectrum

**Figure 2:**
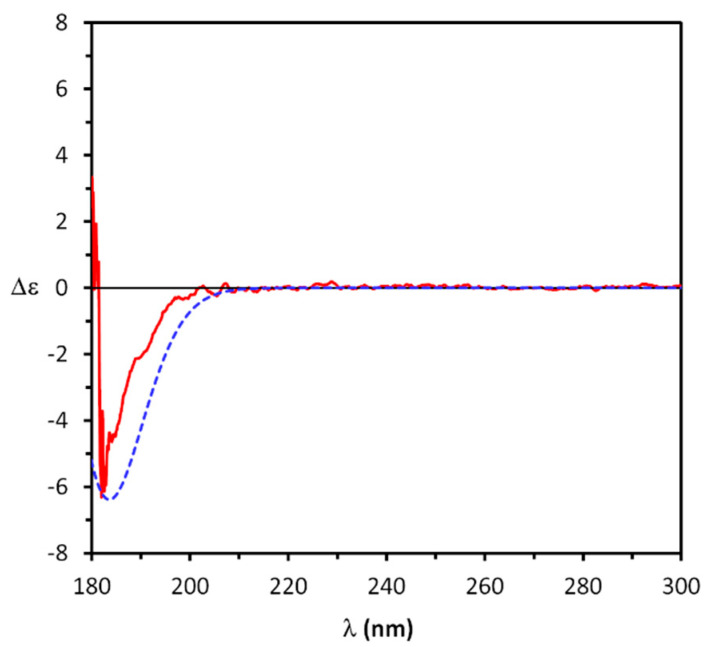
Comparison between experimental ECD spectrum of (-)-**1** (solid red line) and calculated ECD spectrum for (1*S*,2*R*,3*S*,4*R*)-**1** (dotted blue line).

At first, AC assignment to triol (-)-**1** was attempted by comparison of the experimental and the TDDFT-computed ECD spectra, following a reliable and straightforward approach employed for a number of chiral compounds [54], including natural products [55,56]. Accordingly, the UV and ECD spectra of (-)-**1** were recorded in acetonitrile in the 180–300 nm range. As expected, **1** was essentially UV-transparent, while its ECD spectrum (Figure 2) displayed a weak negative structured Cotton effect at about 185 nm, close to the lower limit of accessible spectral window allowed by the CH_3_CN solvent (cut-off 190 nm). Even if the noise of the solvent absorption partly interferes with this signal at shorter wavelengths and does not allow its clear detection, it can be reasonably attributed to a genuine ECD band allied to the n-σ* transitions of the triol hydroxy groups. In fact, chiral hydroxy terpenes were reported to display ECD spectra in solution with a single Cotton effect with a maximum in the 185–190 nm range [57,58].

**Figure 3:**
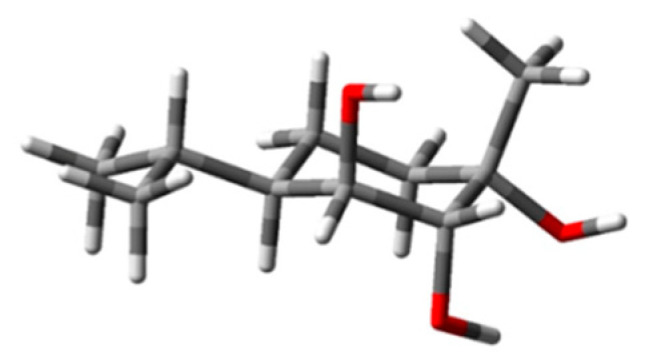
The most stable conformer (DFT/B3LYP/6-31G(d)/IEFPCM(CH_3_CN)) of (1*S*,2*R*,3*S*,4*R*)-**1**. The structure atoms are coloured as follow: carbon (grey), hydrogen (light grey), oxygen (red).

Notwithstanding the uncertainties associated with the ECD signal detection, we proceeded with the AC assignment through the computational approach, with the aim of eventually showing its limitations for this compound. Therefore, computational conformational analysis of **1** was carried out by taking into account the (1S*,2R*,3S*,4R*) relative configuration established by NMR and chemical correlation [34,47]. In this case, the knowledge of the relative configuration permitted the avoidance of carrying out computations on all diastereomers [59], thus limiting the investigation to the two enantiomers of **1**. The conformational analysis was first carried out at a molecular mechanics (MM) level on the chosen theoretical model (1*S*,2*R*,3*S*,4*R*)-**1**. The found conformers were then further optimized at a DFT/B3LYP/6-31G(d)/IEFPCM(CH_3_CN) level of theory. Computations provided 25 appreciably populated conformers for **1**. Among them, 20 conformers, accounting for 87% of the overall population, displayed the isopropyl group at C-4 in an equatorial position and an axial methyl at C-1. Moreover, the seven most populated conformers, accounting for 60% of the overall population, displayed essentially the same conformation of the *p*-menthane ring and of the isopropyl moiety, only differing in the orientation of the OH bonds. As inferred from Figure 3, in these conformers, herein represented by the most populated one (22%), the hydroxy groups at C-1 and C-2 are both in an α position and are parallel to each other, while the OH at C-3 is axial in a β position, pointing on the opposite side of the ring.

UV and ECD spectra for (1*S*,2*R*,3*S*,4*R*)-**1** were calculated on previously found conformers at the TDDFT/CAM-B3LYP/aug-cc-pVDZ/IEFPCM(CH_3_CN) level of theory and averaged over the Boltzmann relative population of the 25 populated found conformers. As inferred from Figure 2, the computed ECD spectrum reproduced the single negative Cotton effect displayed by (-)-**1** in sign and wavelength position, thus confirming the (1*S*,2*R*,3*S*,4*R*) AC for this compound. However, the presence of this single weak band in the ECD spectrum of (-)-**1,** and the fact that this band is interfered with by the solvent absorption, makes such an assignment not fully reliable. The AC assignment of triol (-)-**1** was then carried out by employing the biphenyl chiroptical probe (*vide infra*).

### 3.2. Absolute Configuration Assignment by Application of Biphenyl Chiroptical Probe

**Scheme 1:**
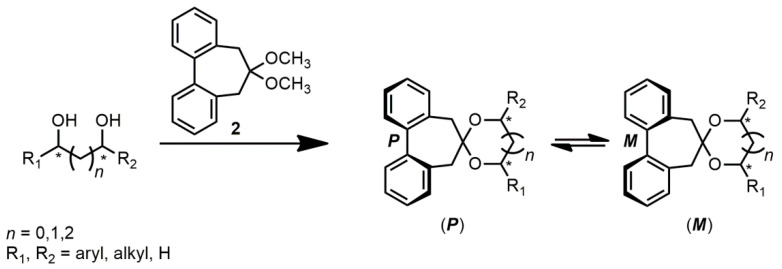
A general scheme for the preparation of biphenyl dioxolanes from diols. The asterisks (*) define the stereogenic centers of the molecule.

The use of biphenyl chiroptical probes was proven to provide a simple, straightforward, and general method for AC assignment to aliphatic non-chromophoric, and then ECD silent, chiral diols, being successfully tested on cyclic and acyclic *threo* [25] and *erythro* [26] diols. According to this approach, diols were transformed into the corresponding biphenyl dioxolanes (Scheme 1), thus obtaining a pair of atropisomeric diastereoisomers with, respectively, a *P* and *M* twist of the biphenyl moiety. The low biphenyl rotational barrier (ca. 14 kcal/mol) [25] allows a thermodynamic equilibrium between the two diastereoisomers; the most stable is then also the most populated.

**Figure 4:**
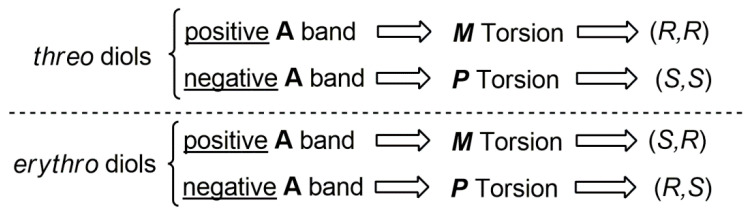
Mnemonic scheme relating the AC of *threo* and *erythro* diols with the sign of the A band (at c.a. 250 nm) in the ECD spectrum of their biphenyl dioxolanes.

The mechanism of chirality induction from the chiral diol to the biphenyl was clarified. It was thus determined that in dioxolanes derived from cyclic and acyclic *threo* (*R*,*R*) 1,2-, 1,3-, or 1,4-chiral diols [60] the most stable diastereomer was the one with an *M* torsion of the biphenyl moiety. The *M* twisted diastereomer was also the most stable in biphenyl dioxolanes derived from (1*S*,2*R*) cyclic *erythro* 1,2 diols. The prevailing sense of twist of the biphenyl moiety is readily revealed by the sign of the ECD Cotton effect at 250 nm, in correspondence to the biphenyl A band absorption [61]. A positive sign of the Cotton effect corresponds to *M* torsion. Reversely, a *P* torsion is allied to a negative A band in the ECD spectrum [62,63,64]. Therefore, by simply looking at the sign of the A band in the ECD spectrum, it is possible to identify the biphenyl torsion and then the AC of the diol inducing the torsion. This finding can then be summarized in the rules reported in Figure 4 which provide a simple guide for the diols’ AC assignment.

**Scheme 2:**
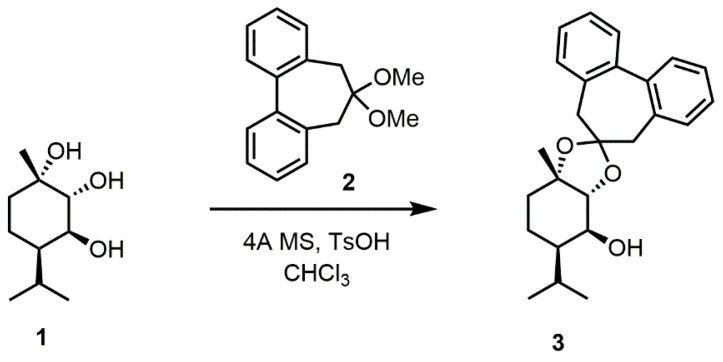
Preparation of the biphenyl dioxolane **3** from triol (-)-**1**.

The flexible biphenyl approach was then applied to (-)-**1** which, following the method described above, was transformed into the corresponding dioxolane **3** by a reaction with dimethylacetal **2** in CHCl_3_, with the presence of traces of *p*-toluene sulfonic acid and use of 4 Å molecular sieves (MS) (Scheme 2). ^1^HNMR analysis shows that the acetal formation occurs between hydroxyl groups at C-1 and C-2 which are *syn* to each other, in agreement with early studies on the acetal hydroxyl protection of compound **1** [47] and the conformer structure in Figure 3, which shows that the formation of the cyclic acetal can only occur between these two hydroxyls.

**Figure 5:**
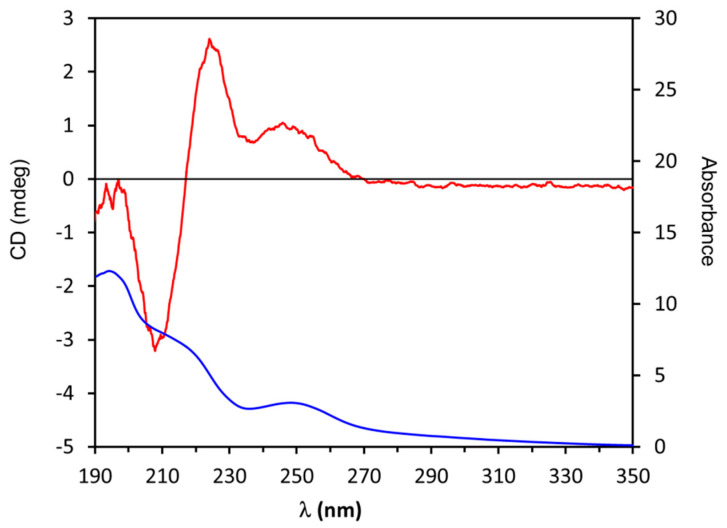
Experimental UV (blue line) and ECD (red line) spectra of **3** in CH_3_CN.

When dealing with natural products, quite often, a very small amount of compounds is available. Therefore, any characterization method needs to be sensitive and, possibly, avoid purification steps. To test the convenience of the biphenyl probe approach for natural products, ECD and UV spectra of compound **3** were directly recorded in the 190–350 nm range, before any purification. In fact, we did not expect significant spectral interference from the possible impurities of unreacted (-)-**1** thanks to its very low UV and ECD response. The UV absorption spectrum of **3** (Figure 5) shows the typical bands of the biphenyl chromophore with the A band clearly visible at 250 nm, a shoulder at 220 nm, and a more intense absorption at 195 nm allied to the biphenyl C band [65]. The ECD spectrum also shows the typical spectral shape observed in diol biphenyl dioxolanes [25,26], displaying an high amplitude band with a positive Cotton effect occurring at 246 nm (i.e., in correspondence to the A band), followed by a positive couplet-like feature centered at 217 nm with sequential positive and negative Cotton effects at 225 nm and 208 nm, respectively. As reported in Figure 4, a positive Cotton effect due to the A band is related to an *M* torsion of the biphenyl aryl–aryl bond which, for an *erythro* diol group like in **1,** leads to the assignment of an (1*S*,2*R*) AC of stereocenters at C-1 and C-2. The knowledge of the relative configuration of the other two stereocenters in **1** also allow the reliable assignment of an (1*S*,2*R*,3*S*,4*R*) AC to diol (-)-**1**.

**Figure 6:**
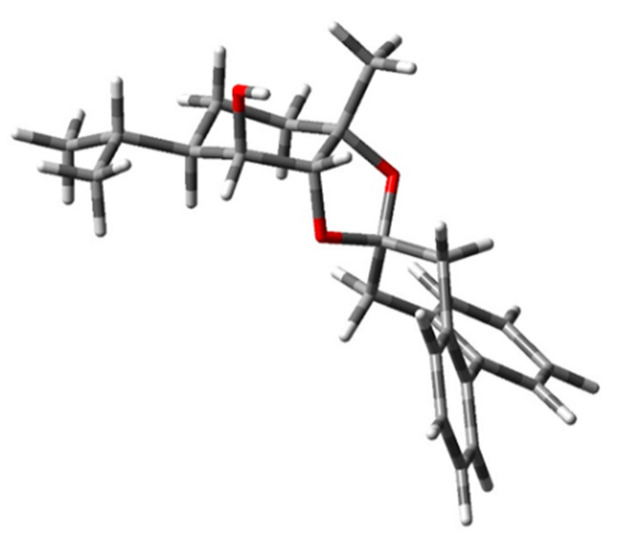
The most stable conformer (DFT/B3LYP/TZVP/IEFPCM(CH_3_CN)) of (1*S*,2*R*,3*S*,4*R*)-**3**. The structure atoms are coloured as follow: carbon (grey), hydrogen (light grey), oxygen (red).

A further confirmation of such an assignment was provided when carrying out a conformational analysis of **3**, first by MM computations and then at the DFT/B3LYP/TZVP/IEFPCM(CH_3_CN) level of theory, providing seven *P* and ten *M* twisted populated atropisomers. DFT computations confirmed the preferred *M* torsion of the biphenyl moiety in the most stable conformer (Figure 6). This agrees with the rule shown in Figure 4. The application of the biphenyl probe approach thus allows for a faster and more reliable AC assignment to triol (-)-**1** with respect to the application of the commoner computational protocol.

## 4. Conclusions

In conclusion, we have herein compared the computational analysis of ECD spectra and the employment of biphenyl chiroptical probes in the AC assignment of (-)-1α,2α,3β-trihydroxy-*p*-menthane (**1**), taken as a representative example of UV-transparent and almost ECD-silent chiral natural products. The (1*S*,2*R*,3*S*,4*R*) AC of (-)-**1**, previously assigned by an empirical chromatographic comparison [48], was herein definitely and non-empirically confirmed by the novel biphenyl approach. This application highlights the usefulness of chiroptical probes in the configurational assignments of natural products and their complementarity to the computational protocols. In fact, the weakness of the ECD spectrum of (-)-**1** makes its computational analysis intrinsically not fully reliable. On the contrary, the biphenyl dioxolane **3** obtained by a simple chemical transformation of this triol shows an intense ECD spectrum, from the signal of which the triol AC can be straightforwardly and reliably established by researchers both with and without advanced expertise in spectroscopy and computations. These results show that the biphenyl probe method can be efficiently and reliably applied to complex molecules, including the natural products, and not only to simple model compounds, constituting a practical tool in the hands of natural product chemists.

## Supplementary Materials

The following are available online at https://www.mdpi.com/article/10.3390/biom12030421/s1. Figure S1: ^1^H NMR Spectrum of (-)-**1**.
