# Supplementary figures and images for: Computational Approaches and Use of Chiroptical Probes in the Absolute Configuration Assignment to Natural Products by ECD Spectroscopy: A 1,2,3-Trihydroxy-p-menthane as a Case Study
Source: Biomolecules. 2022 Mar 9;12(3):421. doi: 10.3390/biom12030421 (PMC8945943; doi:10.3390/biom12030421)

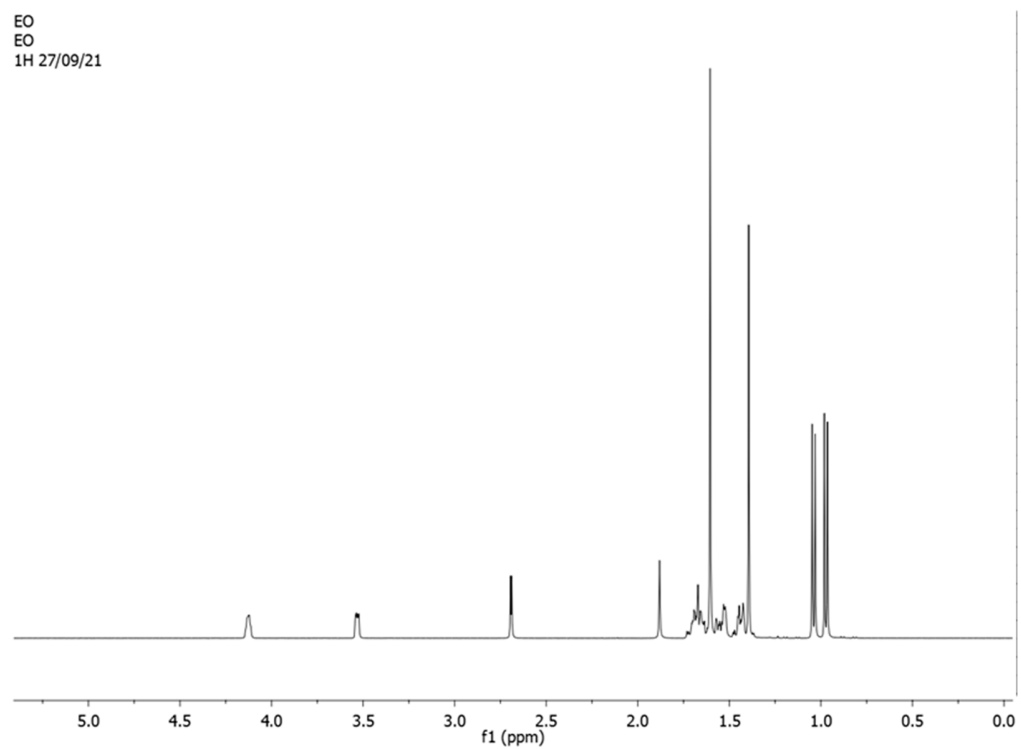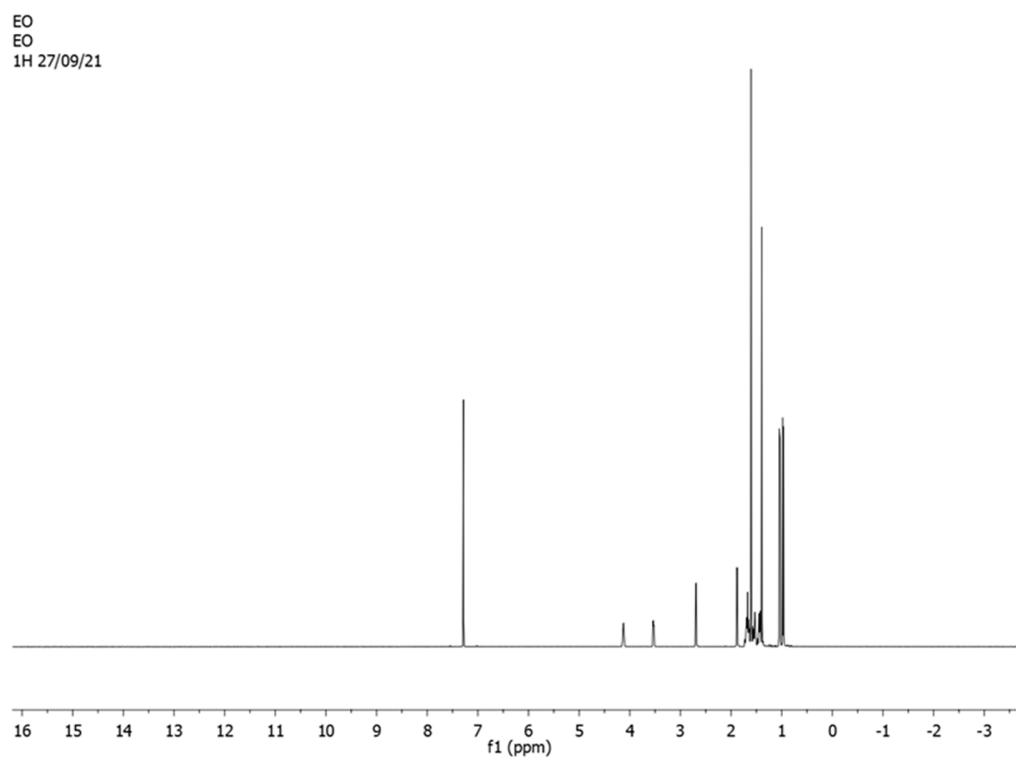

Figure S1. NMR Spectrum of (-)-**1**. Top view expanded spectrum, bottom view full spectrum.

Supplement: Supplementary file 1 [file biomolecules-12-00421-s001.zip › biomolecules-1619754-supplementary.pdf]
